# Transgenic mice with mutations in Nkx2.5 gene: animal model proposal to study non compaction

**DOI:** 10.1186/1532-429X-18-S1-Q34

**Published:** 2016-01-27

**Authors:** Julien Frandon, Stéphanie Bricq, Lucile Miquerol, Monique Bernard, Alain Lalande, Alexis Jacquier

**Affiliations:** 1 Radiology, Grenoble University Hospital, France, Grenoble, France; 2 Le2i, UMR CNRS 6306, Dijon, France; 3 Aix-Marseille Université, CNRS, CRMBM UMR 7339, Marseille, France; 4 Radiology, La Timone, University Hospital, Marseille, France; 5 Aix-Marseille University, CNRS, IBDM UMR 7288, Marseille, France

## Background

The conditional knockout of Nkx 2.5 gene presents hypertrabeculation of the left ventricle (LV) in mice (*Meysen et al., Developmental Biology 2007*). We studied the effects of the deletion of this gene induced at different embryonic days or after birth on the trabeculated mass.

## Methods

We analyzed 17 mice divided in 5 groups : 4 wild mice, 4 heterozygous for NKx2.5 allele, 2 homozygous at D10-11 of embryonic age (trabeculation stage in mice), 4 homozygous at D13-14 of embryonic age (compaction stage in mice), 3 homozygous after birth.

MRI scans were performed 60 days after birth with a preclinical 11.75 T MR system. High resolution cine imaging in small axis view, at the mid base-apex axis was performed. Segmentation of compacted (C) and non-compacted (NC) mass was performed with a semi-automatic software. Papillary muscles were segmented using semi-automatic thresholding and included in the compacted mass. Blood was removed from trabeculae using the same threshold tool.

4 Mice were sacrified and the whole heart was removed and sectioned in the transversal axis. Then immunofluorescence staining was performed on sections at the mid ventricular level corresponding to the image acquired in the short axis by MRI imaging to better delineate C and NC mass. Histological images were manually analysed using ImageJ to validate our method.

All values are presented as median. Interexamination reproducibility was assessed using Bland-Altman analysis (BA) and by computing the correlation coefficient. Differences between groups were assessed using a Kruskall Wallis test or Mann Whitney U test when appropriate. Results were considered significant with a *p* < 0.05.

## Results

### Validation part

NC mass was 1.72 mg for histology and 1.51 mg for MRI (BA: -0.2 ± 0.44 mg).

C mass was 11.93 mg for histology and 13.71 mg for MRI (BA: 1.7 +/- 1.09 mg).

Correlation between MRI and Histologic masses was excellent: r = 0.98, p < 0.01.

### Animal study

In wild mice: NC was 0.07 mg, C was 10,3 mg, NC/C was 0,68%

In transgenic mice: NC was 1.02 mg (p < 0.01), C was 12,24 mg (p = 0.21), NC/C was 7.2% (p < 0.01). Each subgroups have statistically different NC mass (p = 0.047): from 0.56 mg for heterozygous mice to 1.27 g for homozygous at D10-11 of embryonic age.

## Conclusions

Our semi-automatic software is very accurate to evaluate NC mass. Trabeculation seems to increase dramatically when mutation in Nkx2.5 gene is induced early during embryonic development. Our proposal model with mutations of Nkx2.5 gene in mice could help to study the different variants of non compaction.

